# The factor structure of social cognition in schizophrenia: Weak evidence for separable domains

**DOI:** 10.1016/j.scog.2021.100208

**Published:** 2021-07-10

**Authors:** Anja Vaskinn, Kjetil Sundet, Ingrid Melle, Ole A. Andreassen, Svein Friis

**Affiliations:** a Centre for Research and Education in Forensic Psychiatry, Oslo University Hospital, Oslo, Norway; b Norwegian Centre for Mental Disorders Research, Institute of Clinical Medicine, University of Oslo, Oslo, Norway; c Department of Psychology, University of Oslo, Oslo, Norway; d Psychosis Research Section, Oslo University Hospital, Oslo, Norway; e Department of Research and Innovation, Oslo University Hospital, Oslo, Norway

**Keywords:** Theory of mind, Psychosis, Emotion perception, Social perception, Mentalizing

## Abstract

This study examined the factor structure of social cognition in a Norwegian sample of individuals diagnosed with schizophrenia (n = 83). Eight variables from three social cognitive tests from three theoretical domains were included: emotion processing, social perception and theory of mind. Factor analysis with maximum likelihood extraction and oblique rotation resulted in two factors using Kaiser's criterion. Although the two-factor model had better fit than a unifactorial model, it did not represent the data well. Two social cognitive variables did not load on either factor. The two extracted factors did not correspond to an expected distinction between low and high level of processing or between affective and cognitive processes. A non-negligible number of nonredundant residuals between observed and computed correlations suggested poor model fit. In conclusion, this study failed to identify separable dimensions of social cognition in spite of including measures from different theoretical domains.

## 1 Introduction

In spite of its prominence in cognitive research on schizophrenia, the basic structure of social cognition remains unknown. An influential paper on social cognition in schizophrenia, written by experts in the field following a NIMH-sponsored workshop, defined the construct and identified future research needs (Green et al., 2008). Among suggested research areas were investigations of the factor structure of social cognition. Today, almost 15 years later, the field has still not reached a consensus.

Theoretically, social cognition in schizophrenia is usually divided into 4–5 domains (Pinkham, 2014): emotion processing, social perception/knowledge, attributional style, and theory of mind (ToM) (Green et al., 2008). Empirical studies of the factor structure of social cognition have not always produced these domains. In fact, studies have differed, sometimes quite substantially, in the number of identified factors. Some have found two (Buck et al., 2016), others three (Mancuso et al., 2011; Corbera et al., 2013; Mehta et al., 2014) or four (Bell et al., 2009) factors, but there are also reports of one- factor solutions (Browne et al., 2016).

As pointed out (Reidel et al., 2020), these inconsistent findings are partly due to variability in the input to the factor analyses. Examples are the use of different social cognitive tests, different number of tests, tests from different theoretical domains, and differences among participants included in study samples. Replication has been scarce, with some exceptions. In an early study, Mancuso et al. (2011) identified a three-factor structure, consisting of attributional style, along with low- and high-level social cognition. Interestingly, the same research group replicated this three-factor solution in a confirmatory factor analysis using the same social cognitive instruments in a new, but similar sample (Reidel et al., 2020). Of other consistent findings is the tendency for measures of attributional style to load on a separate factor (Buck et al., 2016; Mehta et al., 2014). This aligns with how attributional style associates with other features of schizophrenia, showing non-trivial relationships with positive symptoms (Combs et al., 2009) unlike other social cognitive domains (Ventura et al., 2013). Recently, a two-dimensional model of social cognition was proposed (Etchepare and Prouteau, 2018), with a differentiation between level of processing (low vs. high) and type of processed information (affective vs. cognitive).

Published factor analytic studies have largely been conducted on US samples. We agree with others (Hajdúk et al., 2020) that the research field will benefit from information concerning social cognition in schizophrenia across cultures and languages. Therefore, in this paper, we investigate the factor structure of social cognition in a Norwegian schizophrenia sample, using well-known and validated tests. Our tests represent three theoretical domains (emotion processing, social perception, ToM). Given previous findings of two factors in studies that have excluded the domain of attributional style, we hypothesize that we will find two factors, corresponding to low- (emotion processing) and high- (social perception, ToM) level social cognition.

## 2 Methods

### 2.1 Participants

Eighty-three individuals with a DSM-IV diagnosis of schizophrenia (n = 65) or schizoaffective disorder (n = 18) participated in this study at Oslo University Hospital in Oslo, Norway. The study was approved by the Regional Ethical Committee. All participants provided written informed consent after having received oral and written information about the study (Table 1).

**Table 1:** Demographic and clinical information of the study sample (n = 83).

	Mean	SD
Age (years)	29.5	8.6
Education (years)	12.1	2.5
WASI IQ	100.2	13.4
Males/females (n/%)	54/29	65/35
Illness duration^a^ (years)	7.2	7.2
PANSS positive symptoms	14.2	4.8
PANSS negative symptoms	14.9	5.3

WASI = Wechsler Abbreviated Scale of Intelligence.

PANSS = Positive and Negative Syndrome Scale.

a n = 81.

### 2.2 Social cognitive tests

Three social cognitive tests from three theoretical social cognitive domains (Pinkham, 2014) were administered. *Emotion processing* was assessed with Emotion in Biological Motion (EmoBio) (Heberlein et al., 2004). This is a point-light display task of the ability to perceive emotions in moving bodies. Performance was scored with the proportional method, using Norwegian norms (Vaskinn et al., 2016). In addition to a total score, the EmoBio yields scores for four emotions as well as neutral body movement. The five subscores were used in the analyses (EmoBio happiness, EmoBio sadness, EmoBio anger, EmoBio fear, EmoBio neutral). *Social perception* was measured with the abbreviated Norwegian version of the Relationships Across Domains (RAD) test (Vaskinn et al., 2017); RADshort. *Theory of mind* (ToM) was assessed with the Movie for the Assessment of Social Cognition (MASC) (Dziobek et al., 2006). Differentiation between affective and cognitive ToM has been proposed (Shamay-Tsoory et al., 2007), and the MASC provides scores for both types (MASCaff, MASCcog). We used these two scores in the current study (Table 2).

**Table 2:** Social cognition in the study sample (n = 83).

	Mean (SD)	Min-max
EmoBio sadness	0.83 (0.20)	0–1
EmoBio happiness	0.84 (0.17)	0–1
EmoBio anger	0.75 (0.22)	0–1
EmoBio fear	0.69 (0.32)	0–1
EmoBio neutral	0.86 (0.17)	0–1
RADshort	24.3 (5.8)	0–36
MASCaff	11.7 (2.9)	0–18
MASCcog	17.4 (4.6)	0–26

EmoBio = Emotion in Biological Motion. RAD = Relationship Across Domains. MASC = Movie for the Assessment of Social Cognition. MASCaff = MASC affective ToM. MASCcog = MASC cognitive ToM.

### 2.3 Clinical and cognitive instruments

Psychotic and negative symptoms were measured with Positive and Negative Syndrome Scale (PANSS) (Kay et al., 1987). IQ was assessed with Wechsler's Abbreviated Scale of Intelligence (Wechsler, 2007), 2-subtest version.

### 2.4 Data analyses

All analyses were conducted in SPSS, version 27.0. The 8 social cognitive variables were subjected to factor analysis with maximum likelihood extraction since we wanted to generalize beyond our sample. Number of factors were based on Kaiser's criterion (eigenvalues >1). Factors were rotated with oblique rotation (direct oblimin) as we expected factors to be correlated.

## 3 Results

**Fig. 1:**
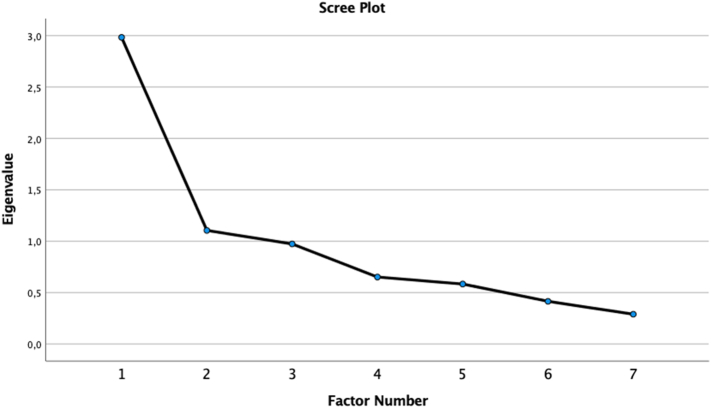
Scree plot.

The statistical analyses revealed that one variable, EmoBio happiness, was weakly associated with the other social cognitive test variables (see Table 3). It was therefore excluded from the factor analysis. The Kaiser-Meyer-Olkin measure indicated sampling adequacy (KMO = 0.737) with individual KMO values for the remaining 7 social cognitive variables ≥0.638. Bartlett's test was significant (<0.001). Five (23%) of the residuals computed between observed and reproduced correlations were nonredundant. One or more of the communality estimates during iterations were >1, indicating that some of the variables had no unique variance. Two factors had eigenvalues >1, explaining 58.4% of the variance. The two MASC variables loaded on the first factor, together with RADshort and EmoBio sadness. EmoBio anger and EmoBio fear loaded on the second factor. EmoBio neutral did not load on either factor. Factor loadings after oblique rotation (direct oblimin) are shown in Table 4. The scree plot (see Fig. 1) was ambiguous, suggestive of one or three factors, in contrast to Kaiser's criterion. We therefore, ran a unifactorial model in order to compare it to the two-factor model. The goodness of fit measures of the two models (two-factor: x^2^ = 12.69, df = 8, *p* = 0.123; unifactorial: x^2^ = 33.02, df = 14, *p* = 0.003) were compared using a chi-square difference test, i.e. by subtracting the chi-square values and degrees of freedom:

$${x^{2}}_{\text{unifactorial}}-{x^{2}}_{\mathrm{two}-\text{factor}}={x^{2}}_{\text{diff}}$$

$${\mathrm{df}}_{\text{unifactorial}}-{\mathrm{df}}_{\mathrm{two}-\text{factor}}={\mathrm{df}}_{\text{diff}}$$

**Table 3:** Correlation coefficients between social cognitive variables.

	EmoBio sadness	EmoBio happiness	EmoBio anger	EmoBio fear	EmoBio neutral	RADshort	MASCaff	MASCcog
EmoBio sadness	1							
EmoBio happiness	0.135	1						
EmoBio anger	0.338	0.110	1					
EmoBio fear	0.121	0.099	0.446	1				
EmoBio neutral	0.357	−0.025	0.172	0.247	1			
RADshort	0.321	0.110	0.462	0.221	0.166	1		
MASCaff	0.378	0.283	0.338	0.143	0.173	0.488	1	
MASCcog	0.459	0.182	0.344	0.188	0.265	0.392	0.685	1

EmoBio = Emotion in Biological Motion. RAD = Relationship Across Domains. MASC = Movie for the Assessment of Social Cognition. MASCaff = MASC affective ToM. MASCcog = MASC cognitive ToM.

**Table 4:** Rotated (oblique) factor loadings based on eigenvalues-greater-than-one extraction criterion.

	Factor 1	Factor 2
MASCcog	**0.891**	0.115
MASCaff	**0.863**	0.105
EmoBio sadness	**0.483**	−0.104
RADshort	**0.413**	−0.280
EmoBio neutral	0.263	−0.075
EmoBio anger	0.046	**−0.875**
EmoBio fear	−0.008	**−0.500**

EmoBio = Emotion in Biological Motion. RAD = Relationship Across Domains. MASC = Movie for the Assessment of Social Cognition. MASCaff = MASC affective ToM. MASCcog = MASC cognitive ToM.

The difference scores (x^2^_diff_, df _diff_) were checked for significance using a standard chi-square table. The values (x^2^ diff = 20.33, df diff = 6) were significant (*p* < 0.005), indicating that the two-factor model had a better fit than the unifactorial model.

## 4 Discussion

This study examined the factor structure of social cognition in a Norwegian schizophrenia sample using three social cognitive tests. Although the factor analysis produced two factors, they were not in line with the expected distinction between lower and higher-level social cognition. It also did not correspond to a differentiation between affective and cognitive content (Etchepare and Prouteau, 2018).

The first factor included the three measures of social perception and ToM, and one EmoBio variable (sadness). This factor seems to reflect higher-level social cognition. The second factor included two EmoBio variables (anger and fear), but generally, the picture for the EmoBio variables was complex. EmoBio happiness and EmoBio neutral appeared to have little in common with the other social cognitive variables as neither could be allocated to a factor. EmoBio fear showed modest associations, except for the stronger association with EmoBio anger. This probably explains why these two variables formed a second factor. EmoBio anger was, however, also strongly associated with the variables in the first factor. It is therefore problematic to include it in a second factor with EmoBio fear.

In fact, we would argue that this study yielded no convincing factor solution. Although comparisons of models indicated that the two-factor model had better fit than a unifactorial model, several concerns remain. Extraction based on Kaiser's criterion (2 factors) was not in agreement with extraction based on the scree plot (1 or 3 factors). There was also a large proportion (23%) of nonredundant residuals between observed and computed correlations, suggesting poor model fit. There were indications during iterations in the statistical analyses, that some variables had no unique variance, and two of the social cognitive variables were not part of the two-factor solution.

The EmoBio test appears to tap into several processes, processes that differ across variables. Some of the EmoBio variables seem not to correspond to the hypothesized low-level social cognition. Lower level social cognition often involves simple decoding of other people's emotions. Perhaps our measure of emotion perception, i.e. point-light displays of human figures moving in a way indicative of a certain emotion, in fact requires drawing inferences more than just the “reading” of emotions. This may be why many of the EmoBio variables indeed were quite strongly related to our measures of social perception and ToM. If we were to offer a speculation, it would be that our dataset and our social cognitive tests largely fall into the higher-level category of social cognition. EmoBio happiness, neutral and fear probably tap into other processes, that are non-shared.

A hierarchical perspective on social cognitive processes, distinguishing between a lower and a higher level of information processing, is described in social neuroscience (Ochsner, 2008). Such a hierarchy is also reflected in dual-process theories of implicit versus effortful processing (Happé et al., 2017), and from a theoretical perspective, a differentiation between implicit, automatic and explicit, culturally learned mind reading has been proposed (Heyes and Frith, 2014). It is possible that many of the tasks used in this study require effortful processing. This, in turn, may be a reason for the not very convincing two-factor solution based on Kaiser's criterion. Future studies should aim to include a range of social cognitive measures involving clear low and high level processes. Method development is still needed, as psychometric challenges have been identified for many social cognitive tests, including the original version of the RAD test used here (Pinkham et al., 2016).

In conclusion, this study failed to provide evidence for the existence of separable dimensions of social cognition, in spite of including tests from three theoretical domains. We encourage further studies on the architecture of social cognition in schizophrenia as this may provide important knowledge for assessment and treatment at the individual level.

## Funding

This work was supported by grants from the 10.13039/501100006095South-Eastern Norway Regional Health Authority (grants #2010007 and #2017069 to AV), the 10.13039/501100005416Research Council of Norway (grant #223273) and Extra Foundation.

## CRediT authorship contribution statement

**Anja Vaskinn:** Conceptualization, Methodology, Data Acquisition, Formal Analysis, Writing – Original Draft, Writing – Review and Editing, Funding Acquisition. **Kjetil Sundet:** Conceptualization, Methodology, Writing – Review and Editing, Funding Acquisition. **Ingrid Melle:** Conceptualization, Methodology, Resources, Writing – Review and Editing. **Ole A. Andreassen**: Conceptualization, Methodology, Resources, Writing – Review and Editing, Funding Acquisition. **Svein Friis:** Conceptualization, Methodology, Writing – Review and Editing, Funding Acquisition.

## Declaration of competing interest

AV has received honorarium from VeraSci. OAA has received speaker's honorarium from Lundbeck and Sunovion and is a consultant for HealthLytix. All other authors declare that they have no conflicts of interests.
